# Patterns of response in patients with advanced melanoma treated with Pembrolizumab (MK-3475) and evaluation of immune-related response criteria (irRC)

**DOI:** 10.1186/2051-1426-2-S3-P103

**Published:** 2014-11-06

**Authors:** F Stephen Hodi, Antoni Ribas, Adil Daud, Omid Hamid, Caroline Robert, Richard Kefford, Wen-Jen Hwu, Tara C Gangadhar, Anthony M Joshua, Peter Hersey, Jeffrey Weber, Richard W Joseph, Hassane Zarour, Roxana Dronca, Linda Gammage, Darcy Hille, Dahai Xue, S Peter Kang, Patrick Chun, Scot W Ebbinghaus, Andrea Perrone, Jedd D Wolchok

**Affiliations:** 1 Dana-Farber Cancer Institute, Boston, MA, USA; 2 University of California, Los Angeles, CA, USA; 3 University of California, San Francisco, CA, USA; 4 The Angeles Clinic and Research Institute, Los Angeles, CA, USA; 5 Gustave Roussy, Villejuif, France; 6 Westmead Hospital and Melanoma Institute of Australia, NSW, Australia; 7 The University of Texas, MD Anderson Cancer Center, Houston, TX, USA; 8 Abramson Cancer Center of the University of Pennsylvania, Philadelphia, PA, USA; 9 Princess Margaret Cancer Centre, Toronto, ON, Canada; 10 University of Sydney, Sydney, NSW, Australia; 11 H. Lee Moffitt Cancer Center, Tampa, FL, USA; 12 Mayo Clinic, Rochester, MN, USA; 13 University of Pittsburgh, Pittsburgh, PA, USA; 14 Merck & Co, USA; 15 Memorial Sloan Kettering Cancer Center, New York, NY, USA

## Background

Unique patterns of response have been observed with immunotherapies. Notably, objective response and prolonged disease stabilization can occur after an initial increase in tumor burden. irRC were developed to better characterize response to immunotherapy based on data for Ipilimumab. We previously showed that patients with melanoma treated with the anti-PD-1 monoclonal antibody Pembrolizumab may also experience unique patterns of response and that conventional response criteria may underestimate the therapeutic benefit of Pembrolizumab [1]. We updated our initial analysis to include an additional 6 months of follow-up.

## Methods

Patients from 3 melanoma cohorts treated with Pembrolizumab 2 mg/kg Q3W, 10 mg/kg Q3W, or 10 mg/kg Q2W in the Phase I KEYNOTE-001 trial served as the source population. Tumor imaging was performed every 12 weeks. Response was assessed by irRC and RECIST v1.1 by independent central review. Patients were managed by irRC by investigator. Early and delayed pseudo-progression were identified using centrally assessed irRC data among patients treated with Pembrolizumab who were followed by imaging for ≥28 weeks as of May 2014. Early pseudo-progression was defined as unconfirmed PD at assessment 1 (ie, week 12) and non-PD at assessment 2. Delayed pseudo-progression was defined as PD at any time point followed by non-PD at the next assessment. Survival data as of May 2014 were analyzed in all 411 patients enrolled in KEYNOTE-001.

## Results

**Figure 1:**
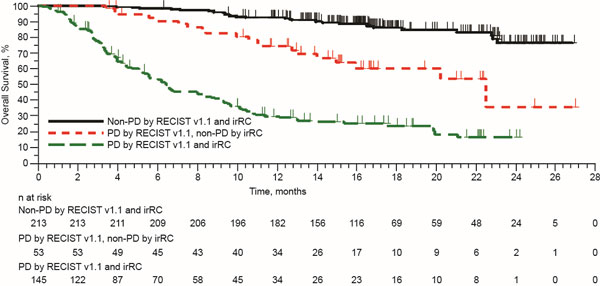
**Kaplan-Meier Estimates of OS**.

Following an incremental assessment of 6 months, there were an additional 16 patients with ≥28 weeks of imaging follow-up (n = 208 total), 1 additional patient who experienced early pseudo-progression (n = 8 total; 3.8%), and 2 additional patients who experienced delayed pseudo-progression (n = 9 total; 4.3%). This corresponded to a 0.9% increase in atypical responses compared with the previous assessment. Based on analysis of best overall response in the total population (n = 411), 2 additional patients had PD by RECIST but CR/PR/SD by irRC (n = 53 total). These 53 patients had favorable OS compared with the 145 patients who had PD by both criteria (Figure 1).

## Conclusions

Pembrolizumab-treated patients with melanoma may experience unique patterns of response and should be managed accordingly. Analysis of OS suggests that conventional RECIST may underestimate the benefit of Pembrolizumab in approximately 10% of patients. These and other data suggest that new standards for response criteria should be considered for PD-1 inhibitors and other immunotherapies.

Registration http://ClinicalTrials.gov, unique identifier NCT01295827
